# Generalized Additive Models Used to Predict Species Abundance in the Gulf of Mexico: An Ecosystem Modeling Tool

**DOI:** 10.1371/journal.pone.0064458

**Published:** 2013-05-14

**Authors:** Michael Drexler, Cameron H. Ainsworth

**Affiliations:** College of Marine Science, University of South Florida, Saint Petersburg, Florida, United States of America; Bangor University, United Kingdom

## Abstract

Spatially explicit ecosystem models of all types require an initial allocation of biomass, often in areas where fisheries independent abundance estimates do not exist. A generalized additive modelling (GAM) approach is used to describe the abundance of 40 species groups (i.e. functional groups) across the Gulf of Mexico (GoM) using a large fisheries independent data set (SEAMAP) and climate scale oceanographic conditions. Predictor variables included in the model are chlorophyll *a*, sediment type, dissolved oxygen, temperature, and depth. Despite the presence of a large number of zeros in the data, a single GAM using a negative binomial distribution was suitable to make predictions of abundance for multiple functional groups. We present an example case study using pink shrimp (*Farfantepenaeus duroarum*) and compare the results to known distributions. The model successfully predicts the known areas of high abundance in the GoM, including those areas where no data was inputted into the model fitting. Overall, the model reliably captures areas of high and low abundance for the large majority of functional groups observed in SEAMAP. The result of this method allows for the objective setting of spatial distributions for numerous functional groups across a modeling domain, even where abundance data may not exist.

## Introduction

The need for ecosystem-based approaches to fisheries management has been widely recognized throughout the world [1]. Marine ecosystem models (e.g., Ecospace, Atlantis, InVitro, OSMOSE, Gadget, IBEM, etc.) are becoming an important tool in achieving those goals as they incorporate predator-prey dynamics and environmental interactions in a spatially explicit context. Spatially explicit models allow managers to better understand certain ecosystem processes, but they require large amounts of data in comparison to models that assume homogeneous space. One example of these additional requirements is that these models require an initial spatial allocation of functional group biomass or abundance. It is not straightforward to develop biomass distribution grids due to the lack of comprehensive stock assessments outside a handful of commercially valued species and there is a particular lack of spatial distribution data from international waters. In most cases, this limits the development of ecosystem models to those areas that are rich in fisheries independent data. Efforts have been made to extrapolate data from limited spatial areas to larger scales using a variety of methods including interpolation over arbitrarily assigned regions [2] and similarity matrices [3]. Generalized additive modeling offers an objective way to predict abundance or biomass according to the known ecology of the animals over broad geographic areas. Generalized additive models (GAMs) [4] are a semi-parametric approach to predicting non-linear responses to a suite of predictor variables. In general, GAMs can be used to identify optimal conditions for a given species using environmental variables (e.g., depth and temperature) in order to predict the likelihood that a given species would inhabit a particular environment, or their abundance [5], [6], [7], [8]. The outputs of these models are often used to interpolate species distributions at high resolution within coarsely sampled areas [9], [5], [7]. Model testing and sensitivity analysis can also help identify influential environmental variables and their corresponding range of influence [9], [5]. In comparative studies, GAMs have often been shown to perform as well or better than other types of predictive models based on environmental conditions [8], [10], [11]. Despite their acknowledgment as a proven tool for ecological analyses, albeit with some caveats [11], few studies, have applied the method to make predictions outside of a sampled area.

Fisheries independent sampling efforts typically result in many zero observations for any given species, particularly in surveys that cover a broad range of habitats or depths. To deal with this problem a number of approaches have been developed to fit these types of data including lognormal delta distributions [12], [13], delta method approximation of variance [14], and zero inflated distributions [15], [16]. All of these methods can be applied to either generalized linear models, or generalized additive models. The latter of the two allows for greater flexibility in the model fitting. Despite these advanced methods dealing with zero inflation, Warton (2005) [17] found that in most cases a negative binomial was sufficient to model data with many zeros. In this paper we utilized a negative binomial GAM to predict the relative abundance of functional groups across shelf areas of the entire Gulf of Mexico (GoM) including Mexican and Cuban waters and areas where fisheries independent surveys do not exist, based on environmental and habitat predictors.

The purpose of this study was to find a single parsimonious framework to predict the distribution of multiple functional groups within an ecosystem. This framework can then be utilized in the objective distribution of relative abundance for a spatially explicit Atlantis ecosystem model of the GoM (Atlantis-GoM) in preparation for NOAA's Integrated Ecosystem Assessment and other applications (Atlantis; [18], [19]). In order to provide better spatial management of fisheries, a better understanding of the ecosystem-wide influences on that stock requires modeling of the entire ecosystem and not just those parts that are adequately sampled. We validate the model by predicting the distribution of pink shrimp (*Farfantepenaeus duroarum*) throughout the Gulf of Mexico and compare the model performance of both the aggregated results used in ecosystem models and the high-resolution gridded values taken directly from the GAM. While numerous species were considered in the model fitting, only the summer abundance of pink shrimp is illustrated here. Pink shrimp were chosen as an example species because they were well represented in the available observational data set used in model training and their distribution is strongly correlated with environmental and habitat predictor variables.

## Methods

### Study Area

**Figure 1:**
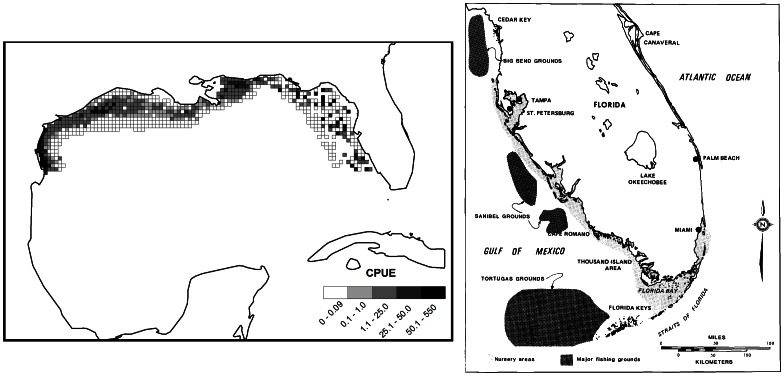
Pink shrimp abundance data. Historical fishing grounds of pink shrimp (*Farfantepenaeus duorarum*) off of the west coast of Florida in 1983 (left) and the observed abundance sampled from SEAMAP sampling locations from 1987 to 2009 in terms of individuals caught per one-hour tow of 40-ft shrimp trawl (right). Reproduced with permission from Bielsa et al. (1983).

The Gulf of Mexico (GoM) is one of the world's 64 Large Marine Ecosystems [20]. This ecosystem spans tropical and subtropical climates and is enveloped by the economic exclusive zones (EEZ) of the United States, Mexico, and Cuba. The EEZ of the United States alone supports 25 million recreational fishing trips [21] and a commercial fisheries harvest in excess of one million tonnes per year [22]. Gulf shrimp remain one of the most important fisheries in the region with combined landings value of 368 million dollars [23]. In this article, we estimate the abundance and spatial distribution of 40 functional groups (groups of species aggregated according to niche similarities). The single-species functional group of pink shrimp is illustrated here in detail as a case study. The range of pink shrimp extends through the entire GoM coastal waters [24]. Nearly 94% of the pink shrimp harvested in the GoM are landed on the west coast of Florida [23] where they are particularly abundant. Known “hot spots” for pink shrimp include the area surrounding the Dry Tortugas as well as the eastern coast of the Golfo de Campeche (Figure 1). The highest adult abundance is found between 9 and 44 m of water [25], [26].

### SEAMAP Groundfish survey

The Southeast Area Monitoring and Assessment Program (SEAMAP) is a multiagency fisheries independent data collection program coordinated by the Gulf States Marine Fisheries Commission [27]. Groundfish surveys are conducted on an annual, and sometimes seasonal, basis using a 40-ft otter trawl throughout the northern GoM. The general area surveyed by SEAMAP includes most the continental shelf up to 200 m depth, but only within the territorial waters of the United States in the northern Gulf of Mexico (Figure 1). Survey data was extracted from the public SEAMAP database [28] and aggregated by functional groups. Sampling effort was estimated as the total area swept of each SEAMAP tow using the Euclidean distance between start and end points and an assumed 40-ft trawling width.

### Environmental Conditions

Predictor variables included in the model were surface chlorophyll *a* (chl *a*), sediment type, bottom dissolved oxygen (DO), bottom temperature, and depth (Table 1). These variables were chosen due to the wide spatial coverage throughout the Gulf of Mexico. Sediment type was divided into the following categories: mud, sand, gravel, and rock. A 0.1^0^ gridded map of seasonal environmental parameters was made for each season (Winter: Jan-Mar, Spring: Apr-Jun, Summer: Jul-Aug, Fall: Sep-Dec) with data collected from the following sources. Measurements of bottom temperature and DO at the maximum depth recorded for each grid point were extracted for each season from the National Oceanographic Data Center (NODC) regional climatology database [29]. Surface chl *a* measurements were obtained by averaging the seasonal composites of MODIS – Terra satellite measurements and the NASA Ocean Biogeochemical Model from 2005–2010 accessed through the GIOVANNI portal (http://disc.sci.gsfc.nasa.gov/Giovanni/overview/index.html). A continuous raster of bathymetry was derived from the SRTM30_PLUS global bathymetry grid [30], which was accessed from the Gulf of Mexico Coastal Ocean Observing System (gcoos.tamu.edu). The best available data on bottom sediment type, dbSEABED2006 [31], [32] does not provide complete coverage for the entire Gulf of Mexico, although the area sampled by SEAMAP has substantial coverage. A nearest neighbor function was executed on a 0.1^0^ grid using the natural neighbor function in GIS v10.0 [33] for those grid points lacking any sediment data. Incomplete and low resolution environmental data was subjected to a spline interpolation using the same grid and GIS software in order to provide a contiguous surface from which to make model predictions. The seasonal environmental conditions grid was then overlaid with the SEAMAP starting locations for a given season. These environmental grids were used both in the fitting of the model and in and in predicting abundance distributions for unsampled areas.

**Table 1:** Data sources for model.

Environmental Parameter	Data Source	Resolution	Manipulations
Abundance	Southeast Area Monitoring and Assessment Program (SEAMAP)	varies	Standardized to area swept centered around each starting point
Surface chlorophyll *a* (chl *a*)	MODIS-Terra 4km Satellite -NASA Giovanni Portal	4km	NA
Sediment	dbSEABED – GoM Data Altas	low	Nearest neighbor interpolation
Bottom Temperature	NODC/GoM regional Climatology	0.1^0^	Spline interpolation
Bottom Dissolved Oxygen (DO)	NODC/GoM regional Climatology	1.0^0^	Spline interpolation
Depth	DOC/NOAA/NESDIS/NGDC – GoM Data Atlas	1.85 km	NA

List of data sources used in the model, the resolution of that data, and any manipulations that were required to attain a contiguous surface with which to make model predictions.

### Model description

A GAM approach was used to predict relative abundance of Atlantis-GoM functional groups in shelf areas across the entire Gulf of Mexico based on estimates of abundance and regional oceanographic conditions occurring at SEAMAP trawl locations. Due to the large number of zero observations and the need for a single parsimonious model to make predictions for a large number of functional groups, a GAM was developed using a negative binomial distribution with an offset for effort [15]. Prior to the fitting of the final model, the data was randomly split into training and test sets: 2/3 of the data was for training and 1/3 for model validation. Once validated, the full set of pink shrimp summer abundance data was then used to fit the model and make the final predictions for the ‘summer’ season. All models were fit using the ‘mgcv’ package in the R version 2.14.0 environment [34] following the equation:
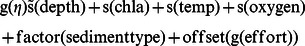
where η represents the expected abundance resulting from the generalization of the predictor terms according to the link function *g*. The abundance data was modeled using a negative binomial distribution with a log link function, including an offset, with equivalent link function, to allow for variations in effort. Function *s* is a thin plate regression spline fit to a given environmental parameter. The smoothness selection was fit using a spline-based penalized likelihood estimation. Theta parameters and weighted penalties were determined by Un-Biased Risk Estimator (UBRE) which is similar to an AIC rescaled [34]. Estimation of the theta parameter was limited to a range of 1–10. An extra penalty was applied to each parameter as the smoothing parameter approached zero, allowing the complete removal of a term from the model when the smoothing parameter is equal to zero. This extra penalty allows for partially automated model selection and is especially useful given the models broad application to numerous functional groups.

The initial training model fits were evaluated by analyzing the total deviance explained and UBRE score. Model performance was evaluated by predicting the abundance at each of the data points in the test data set, given the environmental conditions at that point. The predictions were then compared to the observed values at each respective point. Plots of the predicted vs. observed abundance were made for each group, and a least squares regression was used to evaluate any trends. Once the predictions of the training/test model were compared and deemed suitable for analysis, the entire data set was then used to predict abundance from the environmental conditions within each 0.1^0^ grid cell for depths up to 200 m. To illustrate the usefulness of this method as a way of initializing spatially explicit ecological models, the results of the pink shrimp distributions were averaged according to the Atlantis-GoM spatial polygons and displayed with their associated 95% confidence intervals.

## Results

For pink shrimp, the model described 45.5% (UBRE  = 1.6) of the deviance. The models for the remaining functional groups described between 10% and 83% of the deviance with a median value of 33.6% (Table S1 in File S1). All of the functional groups observed in SEAMAP trawls also had a positive slope of the observed/predicted line indicating that we can reliably predict low and high density areas at least qualitatively. All of the continuous predictors were found to have a smoothing term significantly different from zero (p<0.001) and thus contributed to the model fit for pink shrimp. This was also true for most demersal invertebrate groups. However, the significance of the predictors from other groups not commonly selected by a benthic trawl varied widely.

**Figure 2:**
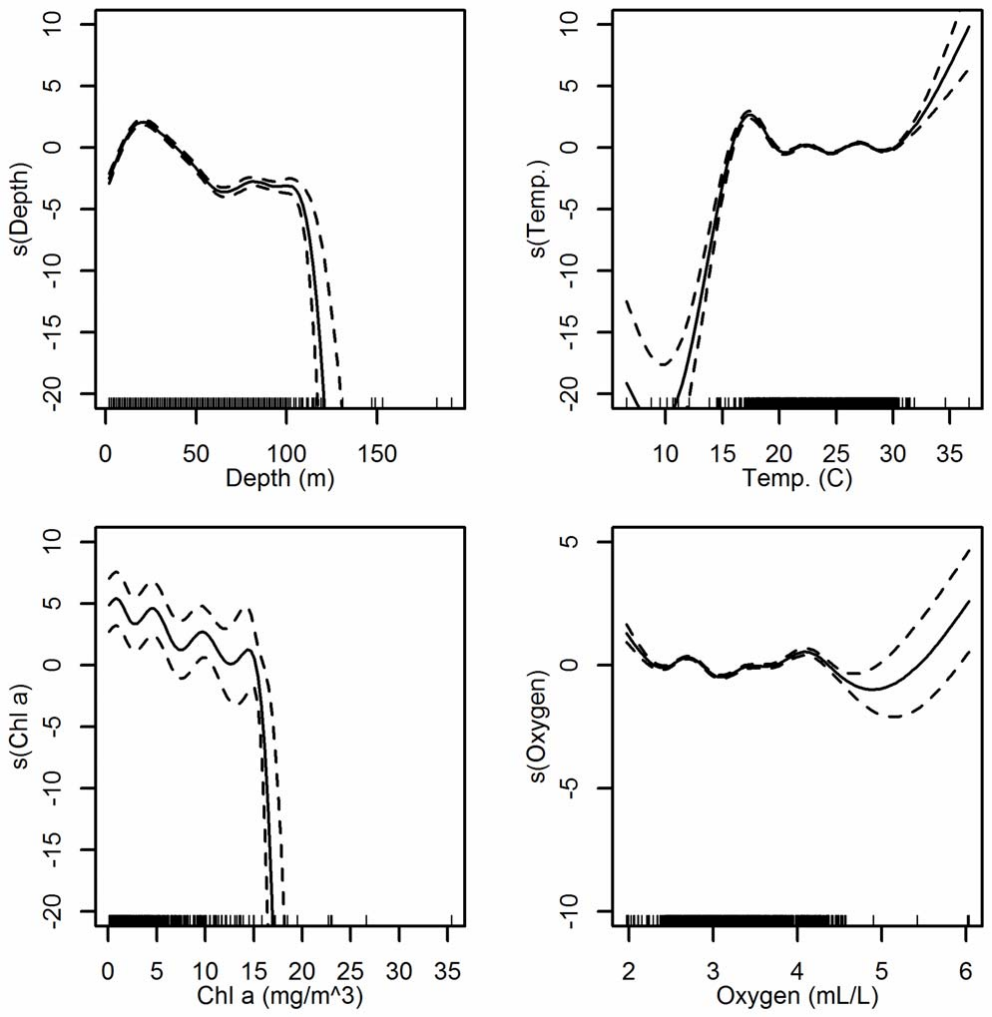
Model fits. Smoothed curve of the additive effect to the estimated abundance of pink shrimp for the individual environmental parameters in the GAM. Dotted lines represent 95% confidence intervals, marks along the lower axis represent a single observation. A straight line represents an additive effect of zero.

Individual parameter values were in general agreement with the habitat suitability index model derived by Mulholland (1984) [24]. Mulholland reports catches of shrimp over a wide range of temperatures (5°–38°C) with the highest density of catch from 20–38°C. The curve fit to the modeled distribution found the highest abundance to be in the range of 18–32°C (Figure 2). Values higher than 38°C likely reflect the error associated with the interpolation of environmental data. Temperatures lower than 15°C had a negative effect on the expected abundance.

**Figure 3:**
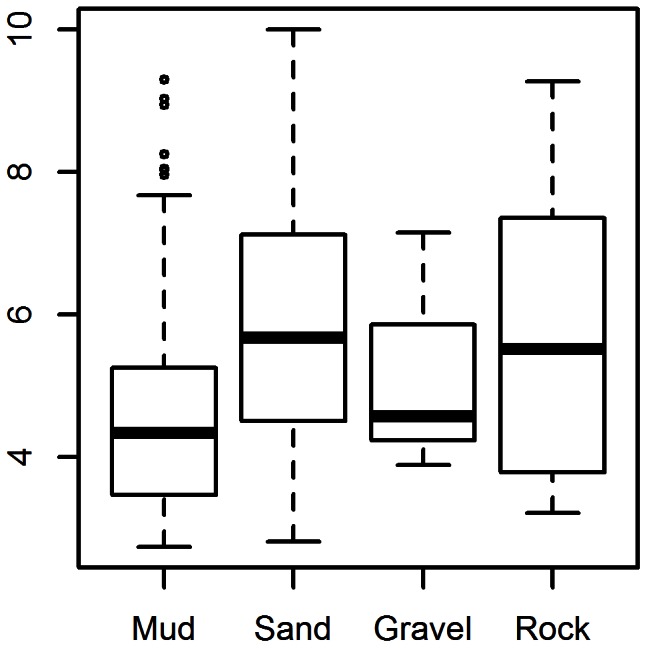
Sediment type vs. pink shrimp abundance. Natural log distribution of pink shrimp abundance for each sediment type. Since we are primarily interested in which category of sediment type is suitable for pink shrimp the zeros have been removed for display purposes.

Mulholland [24] also predicts sandy-silt and silty-sand to be the sediment types with the highest suitability for pink shrimp, followed by hard bottom. The lowest suitability was in soft bottom. The modeled data predicted significantly greater densities of shrimp on sand and rock habitats (p<0.05; Figure 3). Depth only had a slight positive effect on the estimated abundance up to 30–40 m; any depth greater than this had a negative effect on the estimated abundance. Chlorophyll *a* concentrations were inversely correlated to pink shrimp abundance for values up to 15 mg/m^3^ at which point the excepted abundance dropped sharply. The influence of dissolved oxygen did not fluctuate greatly across the range of values between 2.0–4.5 mL/L. Dissolved oxygen values greater than 6 had a positive effect on the model, but were not common in the seasonal averages of the environmental conditions. Therefore, the fitted model reflects previous research pertaining to the habitat preference for pink shrimp with regard to temperature and sediment type and introduces some additional suitability parameters.

**Figure 4:**
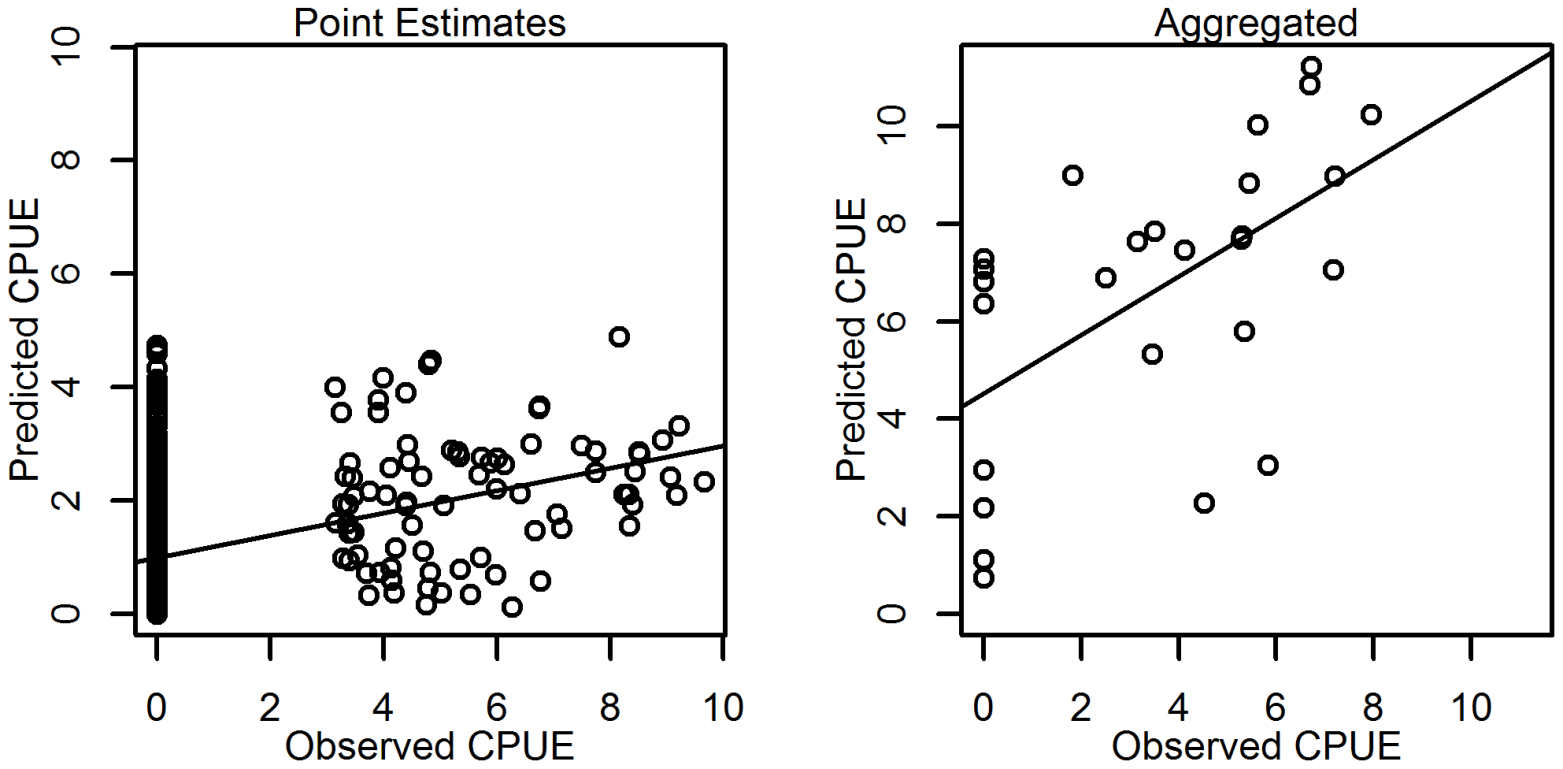
Model performance. Comparison of model predictions to observed ln(abundance) estimated from the environmental conditions of the test data set and compared to the observed values. The resulting scatterplots and least-squared line of fit is shown for both the grid estimates from the model and those estimates aggregated to the Atlantis-GoM polygon level.

Using the test set of SEAMAP data, the ability of the model to predict the response was evaluated. In general, the model predicted a higher mean abundance for those stations where high abundances were observed (Figure 4). Although less than ideal, ideal being equivalent 1∶1 slope, the difference captures the general trend of the data. In every functional group assessed using this model, the slope of the least square line was greater than zero.

The model predictions were then aggregated by polygon and compared to the mean abundance occurring within those polygons with fisheries independent data. The aggregated predictions were in better agreement with the observed data with a normal distribution of residuals around the least square line. However, like the point estimates, the intercept was greater than zero, and may indicate an overestimation of abundances close to zero.

**Figure 5:**
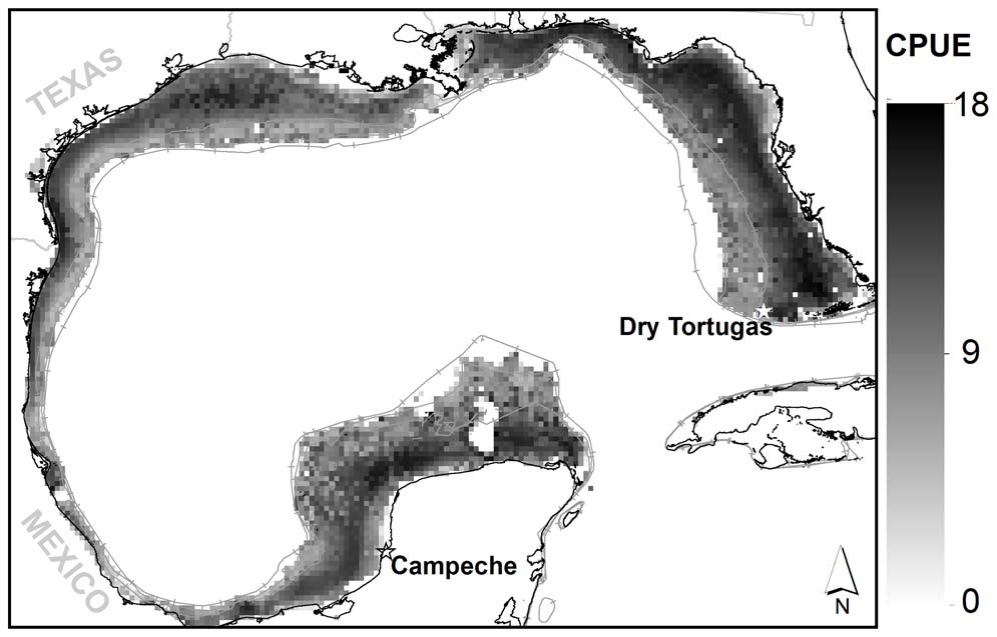
Modeled pink shrimp abundance for the entire GoM. Results of the pink shrimp GAM predicting estimate of abundance. Bathymetric contours of 50 m, 100 m, and 200 m also shown. CPUE expressed as ln(ind./km^2^).

The spatial distribution of the gridded values, predict high abundance along the entire mid-depth portion of the West Florida Shelf, and some additional hotspots near the Dry Tortugas, Louisiana-Texas border, Texas-Mexico border, and on the north-western Campeche Bank (Figure 5). The highest abundance was found in the areas north of the Florida Keys/Dry Tortugas with abundances approaching 1.2 million shrimp per grid cell. Abundances near the Florida panhandle were two orders of magnitude less than those found near the Florida Keys. The hotspots around the Texas-Mexico border and Campeche Bank were a similar order of magnitude less than those near the Dry Tortugas although those distributions were patchy and smaller in area.

**Figure 6:**
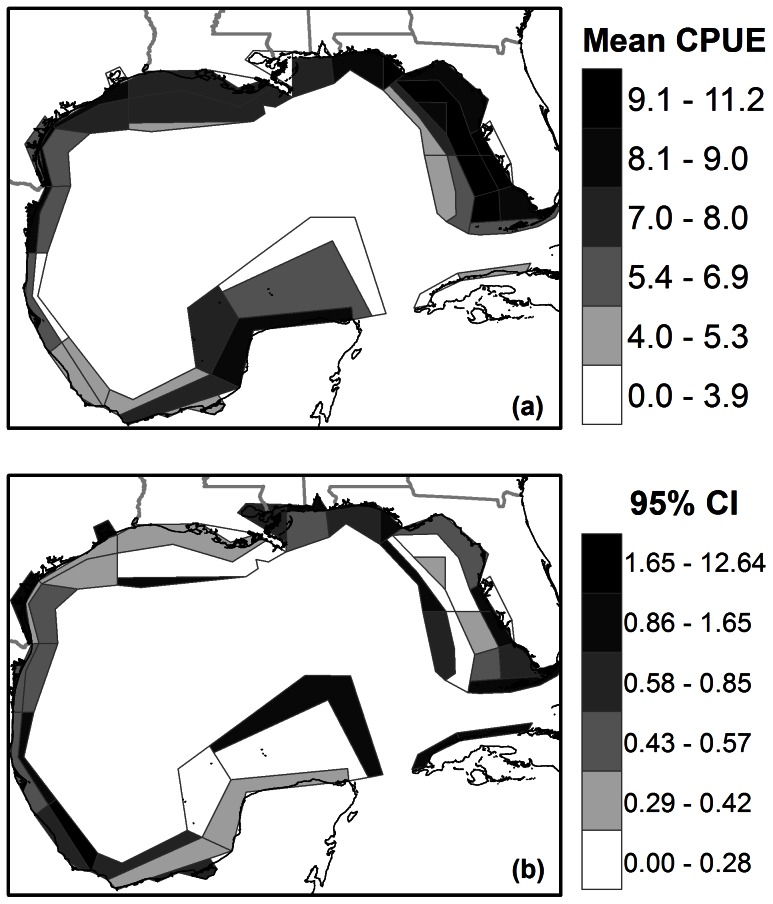
Aggregated model results. Example of the spatial aggregation that can be performed from the GAM predictions of pink shrimp ln(abundance). Mean CPUE ln (ind./km^2^) derived from Figure 6 is calculated according to (a) the box geometry of the Gulf of Mexico Atlantis ecosystem model and (b) the associated 95% confidence intervals (+/− ln(CPUE)) for each box. The few boxes with a confidence interval greater than 1.65 did not have high spatial coverage and the results should be thrown out.

The Atlantis-GoM polygon spatial distributions reflect the general distribution of pink shrimp in the 0.1^0^ gridded results (Figure 5,6). Highest abundance occurs at mid depth over the West Florida Shelf and Dry Tortugas, and the nearshore polygons along the western gulf. The comparison of the observed to predicted values of the aggregated polygons were in better agreement than the higher resolution gridded values (Figure 4). The observed mean of those polygons with SEAMAP data (3.52) was found to be significantly different from the predicted mean (6.62) through a paired t-test (t = 3.90, p = 0.0002). The 95% confidence intervals were inversely related to the mean abundance, and ranged between 0 and 1.65 on a log scale for the majority of the cells. The few polygons with very few data points (estuarine and deep water polygons) had much higher confidence intervals approaching 12 orders of magnitude, and the estimates are not reliable.

## Discussion

The method described here provides a standardized way to generate abundance distributions for models that 1) require comprehensive spatial distributions for 2) a large number of species 3) but are limited in terms of fisheries independent data. Therefore it is an ideal supporting application for spatial ecosystem models. In this paper we extend the use of the GAM approach to make predictions of abundance based on the environmental conditions beyond the sampling domain of the data used to fit the model. We also show how the data derived in this model will be incorporated into the Gulf of Mexico Atlantis ecosystem model in Figure 6. The absolute predictions for individual functional groups may not be precise using the limited abundance data set from SEAMAP employed here. Also, the environmental data used in making predictions is averaged over time and space, missing environmental extremes that may have significant influence on species distributions. Future studies might test environmental data with a higher temporal resolution, given the data is available. Also, the presence of spatial autocorrelation on a regional basis may be addressed by splitting the training/test data into regional blocks and examining the residuals in the remaining sub-regions. In our case we considered this approach impractical due to data scarcity and the application of the modeled results to a course resolution ecosystem model.

The high degree of correlation between model results and the validation data set indicates that we can reliably predict qualitative differences between low- and high-biomass regions, especially in terms of relative abundance. Regardless of the degree of precision provided by this model, the results offer a vast improvement over the assumption of a homogenous distribution of a population commonly used in stock assessments. Further, when aggregated to the level used for spatially explicit ecosystem models, the model provides a better fit to the observed data points. Therefore, the information supplied by this modelling framework can be used to initialize the spatial distribution of species for dynamic ecosystem models whose spatial distributions will settle to a new, but related, equilibrium at run-time. This proof of concept application can be improved as additional CPUE and environmental data become available. Additional data could be incorporated from fisheries-dependent data such as spatially referenced commercial catch statistics and observer data. Coupling these models with spatially explicit estimations of pressures can ultimately determine the absolute contributions of these predictors on species abundance in lieu of pressure.

We demonstrated the utility of the model by predicting areas of high abundance of pink shrimp near the historical fishing areas where no observational abundance data was available. The model results were also in general agreement with previous research regarding the suitability of each parameter used. Thus the extension of this model to the entirety of the southern Gulf of Mexico should provide reasonable estimates of abundance. It should be noted that although pink shrimp occur throughout the Gulf of Mexico, one would expect the species composition of other groups of animals to vary with latitude. While the use of multispecies functional groups occupying similar niches does provide some added flexibility to the final predictions, the inclusion of a latitude parameter could explicitly differentiate these variations in species abundance. Unfortunately, no data is available for latitudes south of Texas-Mexico border to the west, and south of Florida to the east. Thus, including this terms will limit the predictive capabilities to only those latitudes where data exist, undermining the purpose of the model.

While the magnitude of abundance from a benthic trawl may be expected to reflect the absolute abundance of pink shrimp, estimating the abundance of other functional groups, particularly non-demersal and non-benthic species using the same gear will be subject to a catchability bias. There was adequate data to predict the distributions of 40 of the 90 Atlantis-GoM functional groups. Those groups which are less vulnerable to benthic trawling gear, such as large sharks, greater amberjack, king mackerel, spanish mackerel, and squid, did not show a close relationship between observed and predicted abundance values (Figure S1 in File S1). Abundance estimates for these functional groups may be unreliable until additional abundance data from other sampling gear types can be incorporated into the training data set. Abundance was better predicted for slower moving, smaller, mainly demersal species that would be selected by a benthic trawler, such as benthic grazers, bivalves, blue crab, flatfish, pink shrimp, and sessile filter feeders. It is worth re-iterating that predictions of these functional groups were limited by the depth range sampled by SEAMAP trawling. For this reason, the current application should only be used to set distributions on the continental shelf. However, the substitution of a general set of predictors suitable to the pelagic or deepwater environments should yield plausible results.

Despite the positive relationship between the predicted and observed values, none of the least squares lines approached a slope of one. Therefore, the model tends to overestimate abundance where no catch was observed. However, the model did manage to consistently detect a lowered combined abundance at sites where no catch was observed. This bias is likely related to the fact that these simple environmental indices are not satisfactory to explain a portion of the variability in the distribution of these functional groups. The model tends to fail at low population densities that may be heavily influenced by minute differences in oceanographic conditions, patchiness, or other unexplained variability. These differences will not be captured by the regional/seasonal environmental data used in this study. The environmental variables chosen for this study were done so for their wide spatial distribution spanning the entire modelling domain. Given this caveat, these high resolution errors become less important when used to initialize a course resolution model such as the Atlantis-GoM; where polygons are on the order of thousands of square kilometers (Figure 6). The final gridded spatial distributions for all 40 functional groups can be found in Figure S2 in File S1.

Aggregating the results to a courser resolution, i.e. the Atlantis-GoM polygons, allows us to average out the variance, as the large polygons will tend to be closer to the global average. The general distribution of pink shrimp is adequately represented by the Atlantis-GoM polygons (Figures 5, 6). This aggregation provided a better fit to the observational data than the individual points (Figure 4). The significant differences detected between the overall polygon means suggests, as in the gridded model, that we may still be slightly overestimating the total abundance across the entire system. As stated before, this bias may be related to the fact that we are fitting our model to seasonal data. The same reasoning for the bias in the gridded values also applies here. GAMs require many degrees of freedom and introduce a tradeoff between including addition degrees of freedom by aggregating data across seasons or spatial scales, or more highly resolved data with low predictive power to which we chose the prior.

In conclusion, this paper notes the utility of GAMs beyond the common applications of identifying influential environmental variables and interpolating abundance and biomass within sampling regions. For applications like initializing biomass distributions in spatial ecosystem models, where wide spatial and taxonomic coverage is desirable but the benefit of high precision estimates is lost at run-time, these statistical approaches hold unrealized potential.

## Supporting Information

File S1 Table S1, Summary of individual model performance in terms of deviation explained for every Atlantis-GOM functional group observed during SEAMAP sampling from 2005–2010. Figure S1, Combined model fits of the observed (x-axis) versus predicted (y-axis) values of data for all 40 functional groups estimated from this model. The log-log line of least squares is plotted for visualization. Those functional groups with a slope less than or equal to zero (‘deepwater fish’ and ‘large sharks’) are not reliable and should be estimated with a separate set of parameters. Figure S2, Spatial distribution maps predicted by the combined GAM model for all 40 functional groups observed in the SEAMAP database. Grey scale represents the log transformed abundance per square kilometer of each functional group.
(DOCX)
