# Supplementary material for: Generalized Additive Models Used to Predict Species Abundance in the Gulf of Mexico: An Ecosystem Modeling Tool
Source: PLoS One. 2013 May 14;8(5):e64458. doi: 10.1371/journal.pone.0064458 (PMC3653855; doi:10.1371/journal.pone.0064458)

**Table S1.** Summary of individual model performance in terms of deviation explained for every Atlantis-GOM functional group observed during SEAMAP sampling from 2005-2010

| **Functional Group** | **Deviance exp.** | **Functional Group** | **Deviance exp.** |
| --- | --- | --- | --- |
| Benthic Feeding Sharks | 82.7 | Medium Pelagic Fish | 41.7 |
| Benthic grazers | 21.4 | Other demersal fish | 13.9 |
| Bioeroding fish | 61.6 | Other shrimp | 19.3 |
| Bivalves | 73.3 | Pinfish | 31.1 |
| Blue Crab | 29.6 | Pink shrimp | 45.5 |
| Brown Shrimp | 18.0 | Red grouper | 70 |
| Carn. macrobenthos | 21.6 | Red snapper | 22.9 |
| Crabs and Lobsters | 26.1 | Sciaenidae | 26.7 |
| Deep Serranidae | 32.7 | Seatrout | 36.6 |
| Deep Water Fish | 82.1 | Sessile filter feeders | 62.8 |
| Flatfish | 26.2 | Shallow serranidae | 26.1 |
| Gag grouper | 72.9 | Skates and rays | 20.7 |
| Greater Amberjack | 36.3 | Small demersal fish | 23.7 |
| Infaunal meiobenthos | 43.4 | Small pelagic fish | 34.8 |
| Jacks | 16.4 | Small reef fish | 33.6 |
| Jellyfish | 40.1 | Spanish mackerel | 51.1 |
| King mackerel | 33.6 | Spanish sardine | 37.7 |
| Large reef fish | 43.8 | Sponges | 66.1 |
| Large Sharks | 16.3 | Squid | 10.1 |
| Lutjanidae | 45.5 | Vermilion snapper | 72.8 |

**Figure S1.** Combined model fits of the observed (x-axis) versus predicted (y-axis) values of data for all 40 functional groups estimated from this model. The log-log line of least squares is plotted for visualization. Those functional groups with a slope less than or equal to zero (‘deepwater fish’ and ‘large sharks’) are not reliable and should be estimated with a separate set of parameters.

| **Predicted abundnace→** | 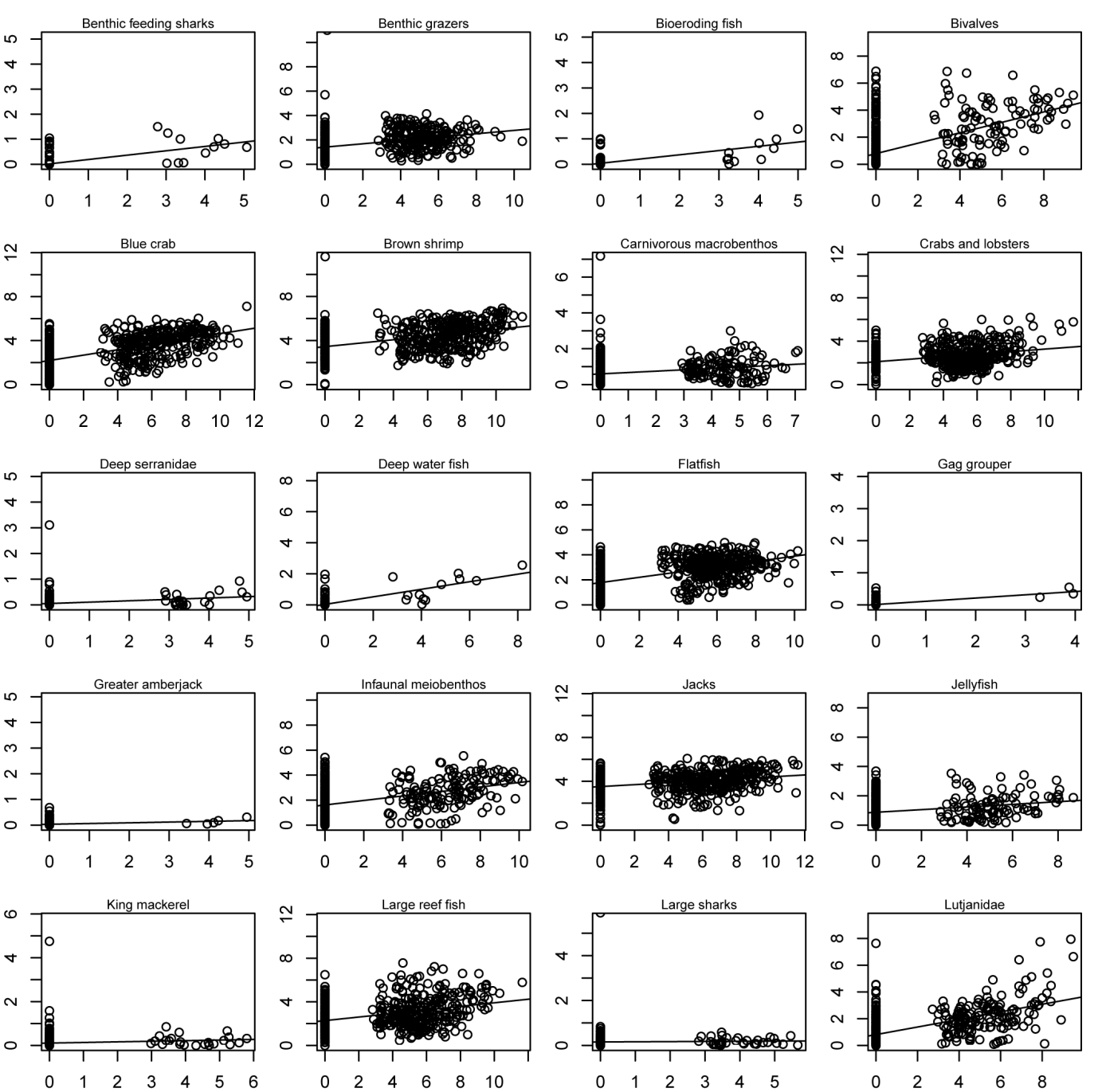 |
| --- | --- |
|  | **Observed abundance→** |

| **Predicted abundnace→** | 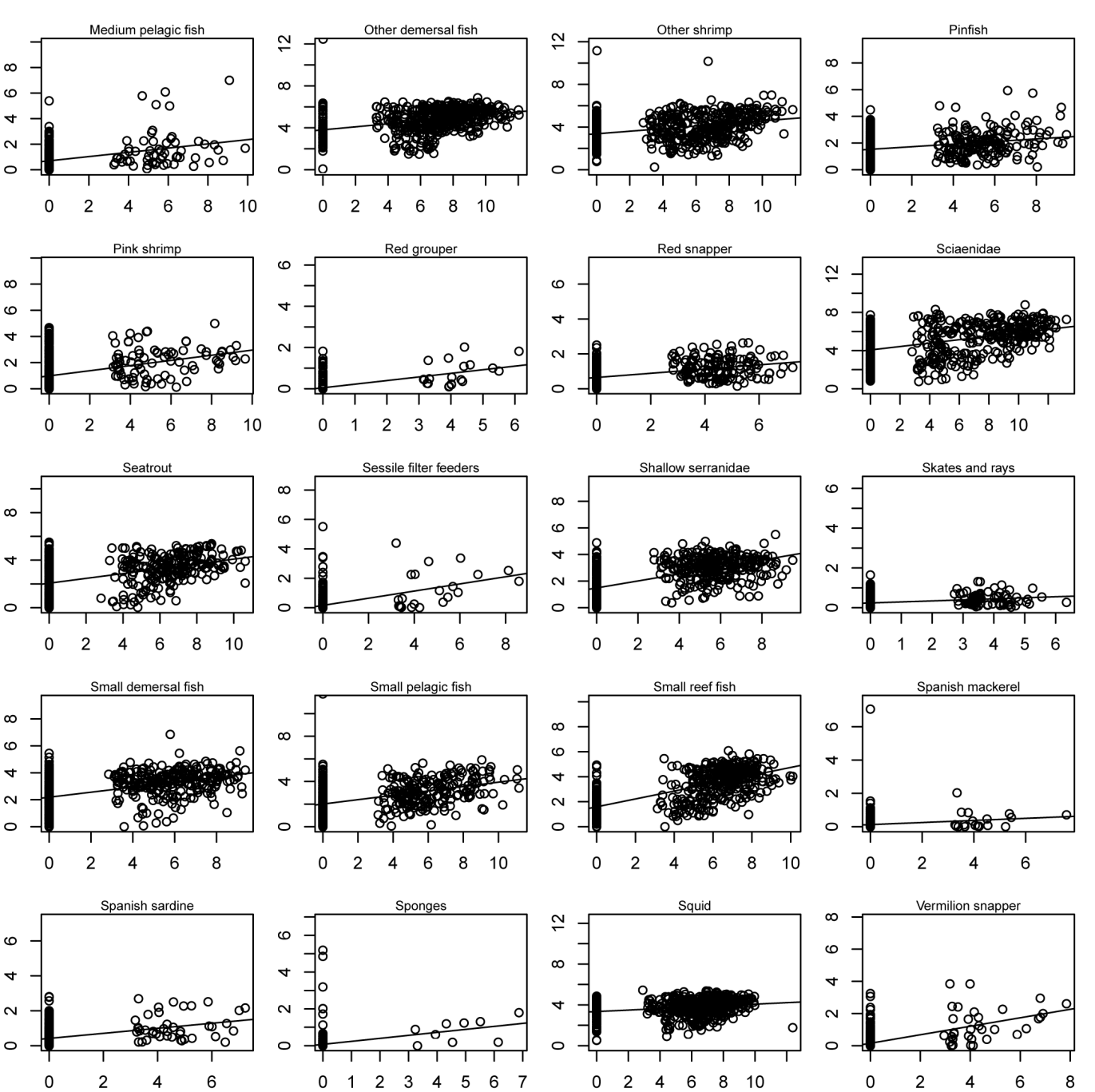 |
| --- | --- |
|  | **Observed abundance→** |

**Figure S2.** Spatial distribution maps predicted by the combined GAM model for all 40 functional groups observed in the SEAMAP database. Grey scale represents the log transformed abundance per square kilometer of each functional group


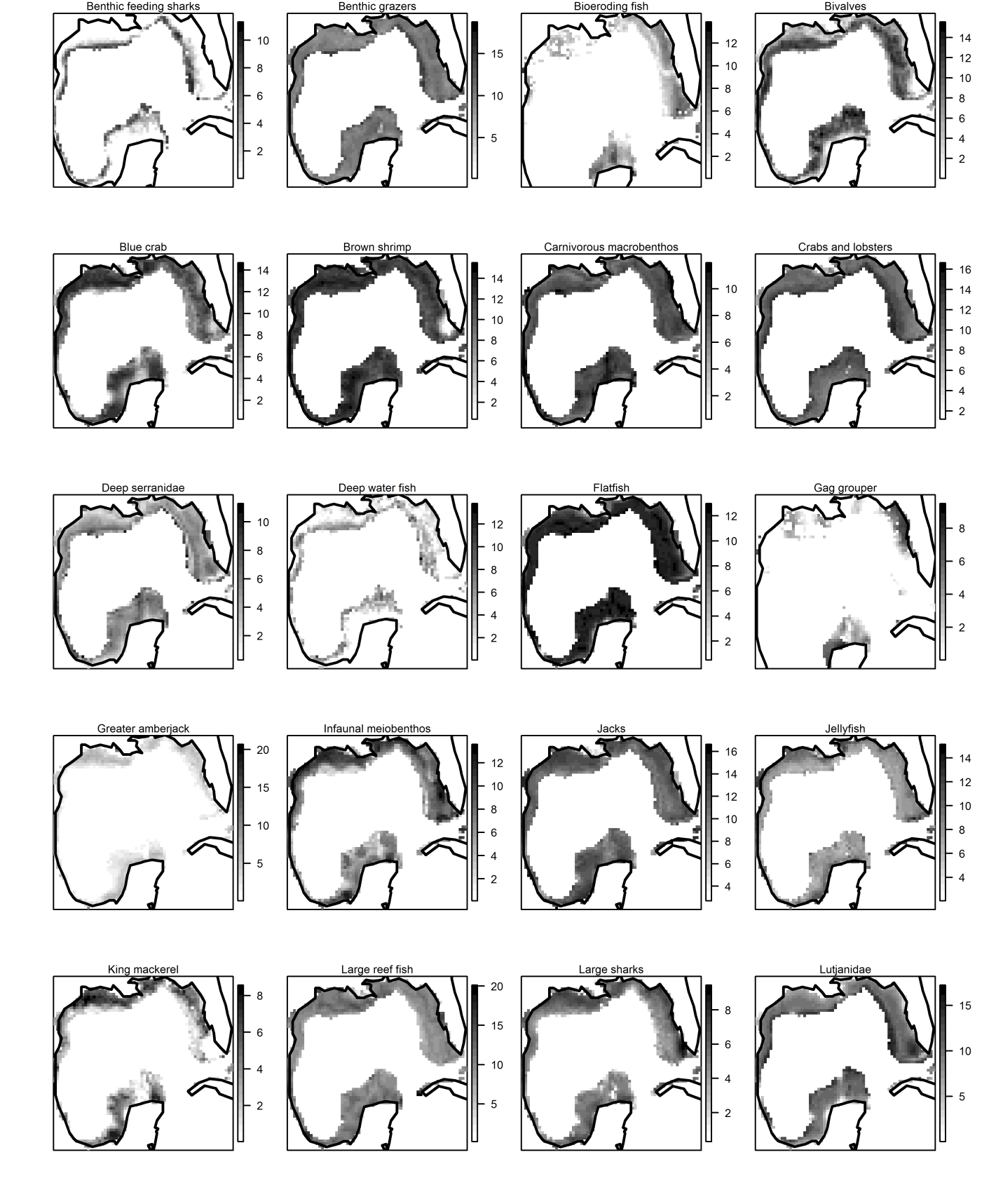


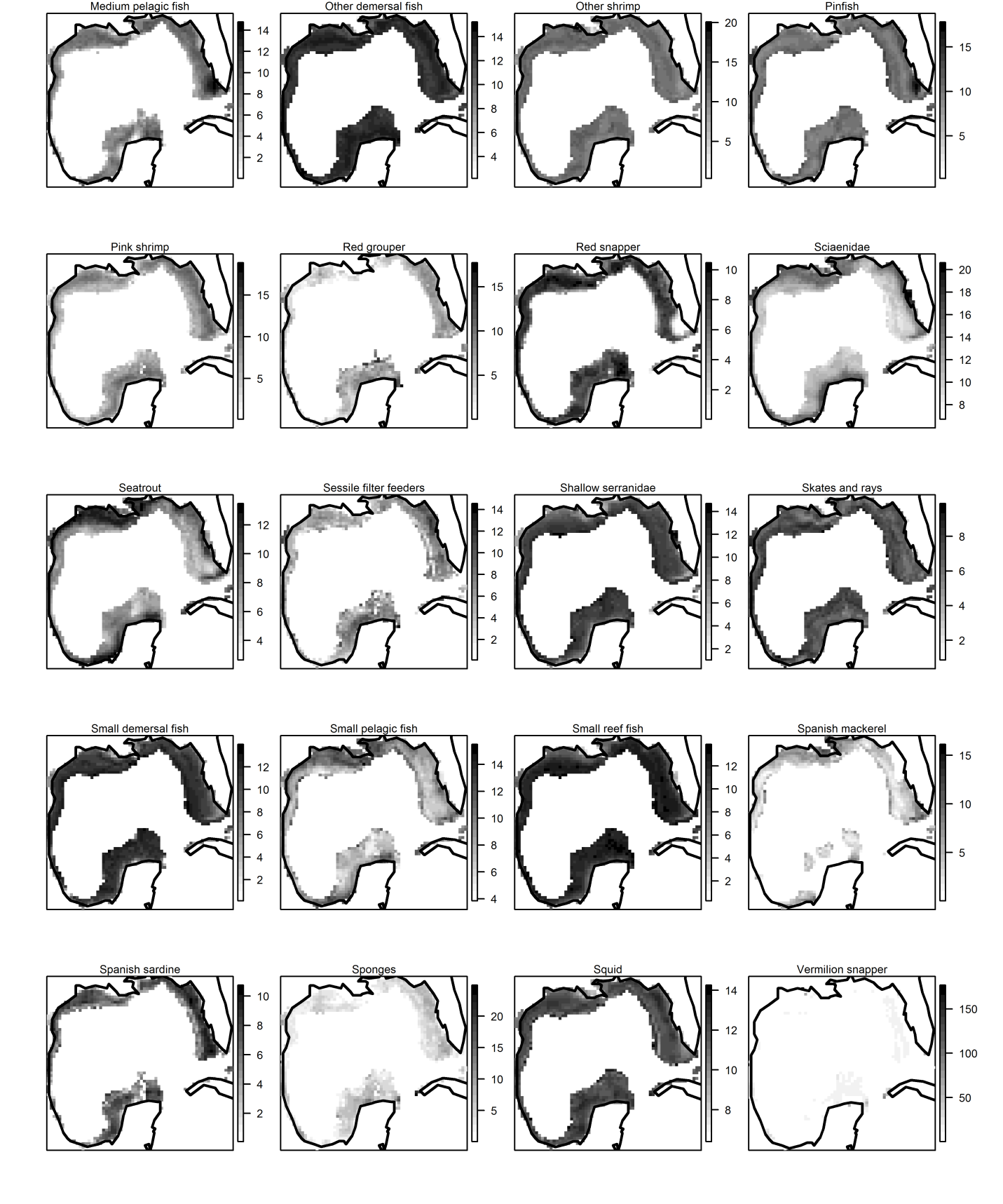

Supplement: File S1 — Table S1, Summary of individual model performance in terms of deviation explained for every Atlantis-GOM functional group observed during SEAMAP sampling from 2005–2010. Figure S1, Combined model fits of the observed (x-axis) versus predicted (y-axis) values of data for all 40 functional groups estimated from this model. The log-log line of least squares is plotted for visualization. Those functional groups with a slope less than or equal to zero (‘deepwater fish’ and ‘large sharks’) are not reliable and should be estimated with a separate set of parameters. Figure S2, Spatial distribution maps predicted by the combined GAM model for all 40 functional groups observed in the SEAMAP database. Grey scale represents the log transformed abundance per square kilometer of each functional group. (DOCX) [file pone.0064458.s001.docx]
